# Sodium selenite mitigates white matter injury after spinal cord injury through GPX4-mediated suppression of ferroptosis

**DOI:** 10.3389/fnmol.2026.1969679

**Published:** 2026-10-29

**Authors:** Jiantao Shi, Linbo Yuan, Hong Su, Wei Quan, Dawei Zhao, Long Wang, Lan Li, Huanran Chen, Jun Zhong, Yi Yin, Shengli Hu

**Affiliations:** 1 Department of Neurosurgery and Key Laboratory of Neurotrauma, Southwest Hospital, Third Military Medical University (Army Medical University), Chongqing, China; 2 Department of Neurosurgery, People's Hospital of Yilong County, Nanchong, Sichuan, China

**Keywords:** ferroptosis, glutathione peroxidase 4, sodium selenite, spinal cord injury, white matter injury

## Abstract

**Introduction:**

Spinal cord injury (SCI) often results in permanent neurological impairments, and preserving residual white matter bundles is critical for sustaining post-injury locomotor function. Oligodendrocytes, the myelinating cells of white matter, are highly susceptible to ferroptosis due to their abundant polyunsaturated fatty acids; however, the role of glutathione peroxidase 4 (GPX4)-mediated ferroptosis regulation in selenium-induced neuroprotection remains unclear. This study aimed to investigate whether sodium selenite (SS) mitigates white matter injury (WMI) and promotes functional recovery via GPX4 upregulation in a rat SCI model.

**Methods:**

*In vivo*, a rat model of SCI was established. Rats were treated with SS alone or in combination with actinomycin D, a classic transcriptional inhibitor known to suppress GPX4 expression. GPX4 expression at the lesion epicenter, oligodendrocyte viability, ferroptosis status, white matter integrity, and locomotor functional recovery were systematically evaluated.

**Results:**

*In vivo*, SS treatment significantly upregulated GPX4 expression at the SCI lesion epicenter (*p* < 0.01). Endogenous GPX4 in oligodendrocytes played a critical role in promoting cell survival and inhibiting ferroptosis in the injured spinal cord. SS administration markedly attenuated WMI and significantly improved locomotor functional recovery in SCI rats (*p* < 0.01). Notably, co-administration of actinomycin D substantially abolished the therapeutic benefits of SS, indicating that GPX4 upregulation is required for SS-mediated neuroprotection.

**Conclusion:**

These findings demonstrate that SS enhances oligodendrocyte survival and mitigates WMI after SCI via GPX4 upregulation, thereby facilitating functional recovery and suggesting SS as a potential therapeutic strategy for SCI-associated neurological dysfunction.

## 1 Introduction

Spinal cord injury (SCI) is a devastating neurological disorder characterized by severe motor, sensory, and autonomic dysfunction, often leading to permanent disabilities that drastically impair patients' quality of life (Ahuja et al., 2017). The pathological progression of SCI encompasses two sequential yet highly interconnected phases: primary and secondary injury. Primary injury results from immediate mechanical trauma, which directly disrupts the structural integrity of axons and myelin sheaths. Subsequently, the secondary injury phase initiates a cascade of pathological events, including sustained neuroinflammation, iron overload, and excessive lipid peroxidation, which collectively contribute to progressive tissue degeneration characterized by astrocyte activation and oligodendrocyte loss (Yuan et al., 2017; Xia et al., 2008; Xia et al., 2015; Li et al., 2014; Hu et al., 2012; Chen et al., 2020; Yao et al., 2019; Feng et al., 2021). Despite substantial progress in elucidating the mechanisms underlying neuronal damage, survival, and regeneration, as well as identifying multiple therapeutic candidates targeting secondary injury, successful clinical translation remains limited and treatment efficacy remains unsatisfactory. These limitations highlight the urgent need to further elucidate the pathogenic mechanisms underlying secondary injury following SCI in order to develop more effective therapeutic strategies.

Notably, accumulating preclinical and clinical evidence indicates that white matter preservation is closely associated with locomotor recovery following SCI (Huang et al., 2023; Duncan et al., 2020; Pukos et al., 2019), and that even a small fraction of spared white matter tracts may be sufficient to sustain meaningful locomotor function (Fehlings and Tator, 1995; Schucht et al., 2002). White matter tracts are predominantly composed of axons ensheathed by oligodendrocytes, whose lipid-rich membranes contain abundant polyunsaturated fatty acids (Shi et al., 2023), rendering these cells particularly vulnerable to lipid peroxidation and ferroptotic cell death (Jhelum et al., 2020). Ferroptosis is a unique form of regulated non-apoptotic cell death characterized by iron-dependent lipid peroxide accumulation and distinct mitochondrial morphological alterations, including mitochondrial shrinkage, reduced cross-sectional area, and increased membrane density (Dixon et al., 2012; Chen et al., 2020; Chen et al., 2021). Importantly, increasing evidence suggests that ferroptosis represents a critical pathological mechanism at the lesion epicenter following SCI, driven by post-traumatic iron accumulation and excessive lipid peroxidation (Chen et al., 2020; Yao et al., 2019), with ferroptotic changes also reported in remote motor-cortex neurons (Feng et al., 2021).

Ferroptosis has emerged as a key contributor to acute central nervous system (CNS) disorders, including stroke (Zille et al., 2017; Alim et al., 2019), traumatic brain injury (Kenny et al., 2019), and intracerebral hemorrhage (Zhang et al., 2018), suggesting that ferroptosis-mediated mechanisms may broadly contribute to progressive tissue damage across multiple CNS pathologies, including SCI. Our previous work has demonstrated that ferroptosis plays an important role in mediating secondary white matter injury (WMI) following SCI. For instance, deferoxamine (DFX), an iron-chelating agent, attenuates ferroptotic injury in rat models of SCI (Shi et al., 2020). Similarly, ferrostatin-1, a well-characterized ferroptosis inhibitor, reduces iron and reactive oxygen species (ROS) accumulation, downregulates ferroptosis-related genes (e.g., IREB2) and proteins (e.g., PTGS2), and inhibits oligodendrocyte ferroptosis (Ge et al., 2022). Furthermore, hepcidin, a key regulator of iron metabolism, promotes oligodendrocyte survival and mitigates WMI by suppressing divalent metal transporter 1 (DMT1) and transferrin receptor (TfR) expression, ultimately improving functional recovery in SCI rats (Shi et al., 2023). Collectively, these findings support targeting ferroptosis as a promising therapeutic strategy for promoting oligodendrocyte survival, preserving white matter integrity, and improving functional recovery following SCI.

Multiple endogenous defense systems have been identified that suppress ferroptosis, among which the glutathione (GSH)-glutathione peroxidase 4 (GPX4) pathway is the most extensively characterized and arguably the most critical for preventing lipid peroxidation. GSH acts as a cofactor for GPX4, which catalyzes the reduction of lipid hydroperoxides (PL-OOH) to non-toxic lipid alcohols (PL-OH), thereby preventing lipid peroxidation and ferroptosis (Conrad and Friedmann Angeli, 2015; Seibt et al., 2019). Other pathways, including ferroptosis suppressor protein 1 (FSP1)-coenzyme Q10 (CoQ10), GTP cyclohydrolase 1 (GCH1)-tetrahydrobiopterin (BH4), and AMPK signaling pathways, also contribute to ferroptosis suppression; however, their specific roles in SCI-associated WMI remain less well defined (Bersuker et al., 2019; Doll et al., 2019; Kraft et al., 2020; Lee et al., 2020). Given the central role of GPX4 in ferroptosis resistance, strategies that enhance GPX4 expression or activity may represent an attractive therapeutic approach for limiting ferroptotic damage after SCI. Notably, selenium has been shown to enhance selenoprotein and GPX4 expression in a mouse model of intracerebral hemorrhage (ICH) through activation of the transcription factors AP-2 gamma and specificity protein 1 (SP1), thereby promoting GPX4-mediated resistance to oxidative damage and suppressing ferroptosis (Alim et al., 2019). These findings suggest that selenium-mediated GPX4 upregulation may provide a mechanistic basis for therapeutic intervention in ferroptosis-related CNS injury.

Selenium is essential for GPX4-mediated ferroptosis inhibition (Alim et al., 2019; Ingold et al., 2018) because GPX4 is a selenoprotein that contains selenocysteine at its catalytic center, making selenium availability a critical determinant of GPX4 expression and activity and, consequently, of cellular resistance to ferroptosis (Yang et al., 2014). As an essential trace element, selenium exerts its biological effects primarily through a diverse family of selenoproteins, including glutathione peroxidases (GPXs), thioredoxin reductase-1 (TXNRD1), and selenoprotein P (SelP). Clinical studies have shown that SCI patients with higher serum selenium levels tend to achieve better neurological recovery, suggesting that selenium may exert neuroprotective effects, attenuate secondary injury, and improve neurological outcomes following SCI (Seelig et al., 2020). These observations raise the possibility that selenium supplementation may represent a therapeutic strategy for limiting secondary tissue damage after SCI. However, the specific role of selenium in attenuating white matter injury (WMI) following SCI, and the underlying mechanisms, particularly whether selenium confers neuroprotection through modulation of ferroptosis and GPX4 signaling, remain poorly understood.

Based on these observations, we hypothesized that sodium selenite (SS), a bioavailable selenium supplement, attenuates oligodendrocyte ferroptosis and subsequent white matter injury (WMI) following SCI through upregulation of GPX4 expression. To test this hypothesis, the present study was designed to address three key questions: (1) Can SS treatment improve locomotor recovery and preserve white matter integrity following SCI? (2) Does SS promote oligodendrocyte survival by suppressing ferroptosis within the injured spinal cord? (3) Is GPX4 upregulation required for the neuroprotective effects of SS after SCI?

## 2 Materials and methods

### 2.1 Animals

All animal experiments were approved by the Ethics Committee of the Southwest Hospital, Third Military Medical University (Army Medical University) (approval no. AMUWEC20210017). A total of 45 adult male Sprague-Dawley rats (200 ± 50 g, aged 4–6 weeks, 39 rats used for experiments and 6 rats died during experiments) were enrolled in this study. Group sample sizes were justified by our published SCI study (Shi et al., 2023). All rats were housed 3 per standard plastic cage under controlled laboratory conditions: a 12-h light/dark cycle, ambient temperature of 22 °C–25 °C, and relative humidity of 55%–60%. Free access to standard rodent chow and sterile water was provided at all times before and after surgery. Rats were purchased from Chengdu YaoKang Biotechnology Co., Ltd. (SCXK2020-034, China). At the experimental endpoint, all rats were deeply anesthetized with 2% isoflurane delivered in an oxygen/air mixture (2–3 L/min). After confirming the complete loss of pain withdrawal reflex, cervical dislocation was performed as a secondary physical method to verify death prior to spinal cord tissue collection.

### 2.2 SS preparation

Sodium selenite (Na_2_SeO_3_) was purchased from Sigma-Aldrich Trading Co., Ltd. (Cat. No. S5261; Shanghai, China). The sodium selenite powder was dissolved in sterile 0.9% saline to prepare a stock solution at a concentration of 10 μM and subsequently diluted to working concentrations of 5 μM and 2.5 μM. The stock solution was stored at −20 °C, whereas working solutions were stored at 2 °C–8 °C and used within 30 days, according to the manufacturer's recommendations to maintain reagent stability and biological activity. The dose of 0.2 mg/kg for oral gavage was selected on the basis of previous studies demonstrating that systemic sodium selenite at this dose exerts neuroprotective effects in rodent models of central nervous system (CNS) injury, including focal cerebral ischemia following intraperitoneal injection in mice (Mehta et al., 2012), and on the established capacity of selenium to support GPX4 expression and ferroptosis resistance in neuronal and genetic models (Alim et al., 2019; Ingold et al., 2018). No signs of selenium-related toxicity were observed in SS-treated rats throughout the experimental period.

### 2.3 Establishment of the rat SCI model

Surgical procedures were performed under sterile conditions as previously described (Shi et al., 2023; Ge et al., 2022). Anesthesia was induced with 5% isoflurane in an oxygen/air mixture (3 L/min) in a closed induction chamber, then maintained at 2% isoflurane via a nose cone (2–3 L/min) while rats were fixed in a stereotaxic frame. Anesthetic depth was confirmed by absent paw withdrawal and corneal reflexes throughout surgery. A 4-cm midline dorsal incision was made, and the paravertebral muscles were bluntly dissected to expose the T9–T11 vertebrae. A laminectomy was performed at the T10 vertebral level to expose the spinal cord while preserving the dura mater. Moderate spinal cord contusion injury was induced by dropping a 20-g weight from a height of 30 mm onto the exposed T10 spinal cord using a 4-mm-diameter impactor tip. The overlying musculature and skin were subsequently closed in layers. Sham-operated rats underwent T10 laminectomy without spinal cord contusion.

Throughout the surgical procedure, body temperature was maintained at 37.0 °C ± 0.3 °C using a feedback-controlled heating pad. Postoperatively, manual bladder expression was performed twice daily until spontaneous voiding resumed. Animals were monitored daily for body weight, hydration status, signs of autophagia, and overall wellbeing, and veterinary care was provided when necessary.

Animals were humanely euthanized if any of the following predefined criteria were met: (1) persistent body weight loss exceeding 20% of baseline values; (2) inability to maintain adequate hydration despite supportive care and repeated bladder expression; (3) severe autophagia or hindlimb ulceration; or (4) complete locomotor loss accompanied by severe distress, including labored breathing or profound lethargy. At experimental endpoints, rats were euthanized with 5% isoflurane in 100% oxygen (3 L/min) in an induction chamber until respiratory arrest. Death was verified by permanent cardiac arrest and pupillary dilation.

### 2.4 Experimental groups

A total of 45 adult rats underwent surgical procedures. Following surgery, six animals were excluded because of unsuccessful SCI induction or unexpected postoperative death. Consequently, 39 rats were included in the final analyses. The sham-operated animals were assigned to a separate control cohort, whereas rats subjected to SCI were randomly allocated to experimental groups using a computer-generated randomization schedule. All treatments were administered once daily throughout the experimental period as follows:

- Sham group (*n* = 6): Rats underwent laminectomy without spinal cord contusion injury and received an equivalent volume of 0.9% sterile saline by oral gavage, matched to the volume administered in the SCI + SS group.
- SCI group (*n* = 11): Rats underwent laminectomy followed by moderate spinal cord contusion injury (see Section 2.3) and received the same volume of 0.9% sterile saline by oral gavage as the Sham group.
- SCI + SS group (*n* = 11): Rats underwent laminectomy and spinal cord contusion injury and subsequently received sodium selenite (SS; 0.2 mg/kg body weight) by oral gavage once daily.
- SCI + SS + Act-D group (*n* = 11): Rats underwent laminectomy and spinal cord contusion injury and received combined treatment with SS (0.2 mg/kg body weight, oral gavage) and actinomycin D (Act-D; 1 μg/g body weight, intraperitoneal injection). Both agents were administered once daily at separate time points to minimize potential drug interactions. Act-D is a classical transcriptional inhibitor, and the dose of 1 μg/g (equivalent to 1 mg/kg) was selected based on previous *in vivo* rodent studies in which intraperitoneal administration of Act-D at 1 mg/kg inhibited transcription-dependent responses (Rosenblum et al., 1995). Act-D was co-administered with SS to block the de novo transcription of GPX4, thereby testing whether SS-induced GPX4 upregulation is required for the neuroprotective effects of SS.

### 2.5 BBB locomotor rating score

Hindlimb locomotor function was evaluated using the Basso–Beattie–Bresnahan (BBB) locomotor rating scale, a widely validated 21-point behavioral assessment tool for experimental SCI (Basso et al., 1995). BBB scores range from 0 (complete hindlimb paralysis) to 21 (normal locomotor function) and assess multiple aspects of locomotor recovery, including joint movement, weight support, stepping ability, interlimb coordination, and trunk stability. All behavioral assessments were conducted in a quiet testing room under standardized lighting conditions. All BBB scoring was performed during the light phase of the 12 h light/12 h dark cycle (8:00–18:00). Each rat was placed individually in a circular open-field arena (120 cm in diameter and 40 cm in height) and allowed to acclimate for 2 min before assessment. Locomotor activity was then observed and recorded for 5 min using a high-definition digital camera positioned above the arena for subsequent analysis. BBB scoring was independently performed by two investigators blinded to experimental group allocation. Scores were assigned based on hindlimb joint movement, weight-bearing ability, stepping frequency, interlimb coordination, and tail position. When the scores assigned by the two observers differed by ≥2 points, the corresponding video recordings were jointly reviewed until a consensus score was reached. The mean score from the two observers was used as the final BBB score for statistical analysis. Behavioral assessments were performed at 1, 7, 14, 21, and 42 days post-injury (dpi). All evaluations were conducted in accordance with the standardized protocol described by Basso et al. (1995), with testing conditions maintained consistently throughout the study.

### 2.6 Luxol Fast Blue (LFB) staining

Luxol Fast Blue (LFB) staining was performed to evaluate white matter preservation and myelin integrity at 14 days post-SCI according to a modified protocol described previously (Kozlowski et al., 2008). Transverse sections of the T10 spinal cord encompassing the injury epicenter (*n* = 7 per group) were fixed in 4% paraformaldehyde (PFA) in 0.01 M phosphate-buffered saline (PBS, pH 7.4) at 4 °C for 24 h and subsequently embedded in paraffin. Serial transverse sections (5 μm thick) were cut, mounted on poly-L-lysine-coated slides, and baked at 60 °C for 2 h to enhance tissue adhesion. For LFB staining (Luxol Fast Blue, tar purple method), sections were deparaffinized in xylene, rehydrated through a graded ethanol series, and rinsed in distilled water. Sections were then immersed in 95% ethanol for 2 min and incubated in LFB solution (Solarbio, Cat. no. G3245) at room temperature for 16–18 h. Excess stain was removed by washing in 95% ethanol, followed by a brief rinse in distilled water. Sections were subsequently differentiated in Luxol differentiation solution, with differentiation monitored microscopically until a clear distinction between gray and white matter was achieved. The differentiation reaction was terminated by thorough rinsing in distilled water. Sections were then dehydrated through 95% and 100% ethanol, cleared in xylene, and coverslipped with neutral balsam. LFB-stained sections were examined under bright-field microscopy. Intact myelin was identified by intense blue staining, whereas non-myelinated or demyelinated regions exhibited reduced or absent staining and appeared colorless to pale blue.

### 2.7 Immunohistochemistry (IHC)

At 14 days post-injury (dpi), immunohistochemical staining was performed on paraffin-embedded spinal cord sections. Sections were deparaffinized in xylene, rehydrated through graded ethanol solutions, and subjected to antigen retrieval by heating in 0.01 M citrate buffer (pH 6.0) for 15 min, followed by cooling to room temperature (RT). Endogenous peroxidase activity was blocked with 3% hydrogen peroxide (H_2_O_2_) for 10 min at RT, and sections were subsequently washed three times with phosphate-buffered saline (PBS). To minimize non-specific binding, sections were incubated in 5% bovine serum albumin (BSA) prepared in PBS containing 0.5% Triton X-100 (Sangon Biotech, China) for 1 h at RT. After blocking, sections were incubated overnight at 4 °C with anti-CC1 primary antibody (RRID:AB_2834125), which recognizes the RNA-binding protein Quaking 7 (QKI-7) in mature oligodendrocytes (Bin et al., 2016), diluted in 1% BSA/PBS. The following day, sections were washed three times with PBS containing Tween-20 (PBS-T) and incubated with horseradish peroxidase (HRP)-conjugated goat anti-rabbit IgG secondary antibody (Beyotime, Beijing, China). After additional PBS-T washes, immunoreactivity was visualized using a 3,3′-diaminobenzidine (DAB) detection kit according to the manufacturer’s instructions, with color development monitored microscopically. Sections were then counterstained with hematoxylin for 30 s, differentiated in 1% hydrochloric acid–ethanol solution for 5 s, and rinsed under running water for 10 min to achieve nuclear bluing. Finally, sections were dehydrated through graded ethanol solutions, cleared in xylene, and coverslipped with neutral balsam. Images were acquired using a Leica bright-field microscope (Leica Microsystems, Germany). For quantitative analysis, six sections per animal and four non-overlapping fields per section were analyzed. CC1-positive cells were quantified and averaged for each animal. All image acquisition and analyses were performed by an investigator blinded to experimental group allocation. Results are presented as mean values for each group.

### 2.8 Immunofluorescence (IF)

Immunofluorescence staining was performed on spinal cord sections collected at 14 days post-injury (dpi) (*n* = 9). Sections were fixed in 4% paraformaldehyde (PFA) in 0.01 M phosphate-buffered saline (PBS; pH 7.4) for 30 min at room temperature (RT), washed three times with PBS to remove residual fixative, and permeabilized with 0.3% Triton X-100 (Sangon Biotech, China) in PBS. To minimize non-specific binding, sections were blocked with 5% bovine serum albumin (Sigma-Aldrich, Cat. no. A7906) in PBS for 1 h at RT in a humidified chamber. After removing blocking solution, sections were incubated overnight at 4 °C with primary antibodies diluted in 1% BSA-PBS: anti-GPX4 (RRID:AB_2909469), anti-dMBP (RRID:AB_2140351), anti-CNPase (RRID:AB_10861310), and anti-MBP (RRID:AB_2665438). The next day, sections were washed 3× with 0.1% Tween-20-PBS (5 min each), then incubated with species-matched secondary antibodies (FITC-conjugated goat anti-mouse IgG and Cy3-conjugated goat anti-rabbit IgG; Abcam, UK) in 1% BSA-PBS for 2 h at RT in the dark. After 3× PBS-T washes, nuclei were counterstained with DAPI (Beyotime, China) for 10 min at RT, followed by three final PBS washes. Sections were mounted with anti-fluorescence quenching medium. Fluorescence images were acquired using a Leica SP8 confocal laser scanning microscope (Leica Microsystems, Germany) and analyzed using ZEN 2011 software (Carl Zeiss, Germany). At least five non-overlapping fields were randomly selected from each section under identical imaging settings for quantitative analysis.

### 2.9 Western Blot (WB)

At 14 days post-injury (dpi), T10 spinal cord tissue samples (*n* = 12), encompassing the injury epicenter and adjacent spinal cord tissue (0.5 cm in length; 0.25 cm rostral and caudal to the lesion center), were rapidly harvested and homogenized for protein extraction. Total protein concentrations were determined using a bicinchoninic acid (BCA) protein assay kit (Takara, Cat. No. T9300A). Equal amounts of protein (50 μg per sample) were separated by 10% sodium dodecyl sulfate-polyacrylamide gel electrophoresis (SDS-PAGE) under reducing conditions and subsequently transferred onto polyvinylidene fluoride (PVDF) membranes (Roche, Indianapolis, IN, USA). Membranes were blocked with 5% (w/v) non-fat milk prepared in Tris-buffered saline containing 0.1% Tween-20 (TBST) for 2 h at room temperature (RT). To facilitate the detection of proteins with similar electrophoretic mobility, membranes were sectioned according to molecular weight based on a 180-kDa prestained protein marker (Nordzane, China). Membranes were then incubated overnight at 4 °C with the following primary antibodies: anti-SMI-32 (RRID:AB_3070332), anti-degraded myelin basic protein (dMBP) (RRID:AB_2140351), anti-iron regulatory protein 2 (IRP2) (RRID:AB_3073421), anti-GPX4 (RRID:AB_2909469), and anti-GAPDH (rabbit; Huaan, China). Following primary antibody incubation, membranes were washed with TBST and incubated with the appropriate horseradish peroxidase (HRP)-conjugated secondary antibodies at room temperature. Immunoreactive bands were visualized using Western Bright enhanced chemiluminescence (ECL) reagents and a Tanon-4200 imaging system (Tanon, China). Band intensities were quantified by densitometric analysis using Image Lab software (Bio-Rad, CA, USA), and target protein expression levels were normalized to GAPDH. GAPDH was selected as the internal loading control because its expression remained stable and comparable across all experimental groups in the present study (no statistically significant intergroup differences, *P* > 0.05), consistent with its routine and validated use as a loading control in western blot analyses of rodent spinal cord injury models (Shi et al., 2020; Ge et al., 2022; Shi et al., 2023). In addition, protein loading was standardized by BCA-based quantification (50 μg per lane).

### 2.10 Transmission electron microscopy (TEM)

Transmission electron microscopy (TEM) was performed at 14 days post-injury (dpi) to evaluate myelin ultrastructure, as previously described (Shi et al., 2020; Yao et al., 2019), and mitochondrial morphology, as previously described for ferroptotic mitochondria (Zille et al., 2017). Briefly, spinal cord tissue samples encompassing the lesion epicenter and adjacent spinal cord tissue (2 mm in length; 1 mm rostral and caudal to the lesion center) were collected following transcardial perfusion and initially fixed in 1.25% glutaraldehyde overnight. The samples were subsequently maintained in fresh 1.25% glutaraldehyde for an additional 3 days at 4 °C. After fixation, specimens were washed with buffer and post-fixed in 1% osmium tetroxide (OsO_4_) for 2 h at room temperature. Samples were then stained with uranyl acetate, dehydrated through a graded acetone series, infiltrated with propylene oxide, and embedded in epoxy resin. Ultrathin sections were prepared using an ultramicrotome (UC7, Leica, Vienna, Austria) and examined with a Hitachi HT7700 transmission electron microscope (Hitachi, Tokyo, Japan). For quantitative analysis, at least three independent samples per group were evaluated by investigators blinded to experimental group allocation. Myelin sheath thickness and the proportion of morphologically abnormal mitochondria (including shrunken mitochondria with increased membrane density, a characteristic feature of ferroptosis) were quantified using ImageJ software (Version 1.8, NIH, Bethesda, MD, USA). For the assessment of mitochondrial morphology, electron micrographs were acquired from oligodendrocytes located within the white matter of the lesion epicenter. Oligodendrocytes were identified by their characteristic ultrastructural features, including electron-dense cytoplasm, abundant microtubules and rough endoplasmic reticulum, and the continuity of their processes with myelinated axons.

### 2.11 Statistical analysis

Statistical analyses were performed using GraphPad Prism 9.0 software (GraphPad Software Inc., San Diego, CA, USA). Data are presented as mean ± standard deviation (SD) unless otherwise specified. Normality of data distribution was initially assessed using the Shapiro–Wilk test. Owing to the limited sample size of *in vivo* animal experiments, this test had insufficient statistical power to generate reliable W statistics and exact *P* values. In accordance with standard practice in preclinical neuroscience research, data were assumed to follow a normal distribution, and parametric statistical tests were applied. Homogeneity of variances was confirmed by the *F* test for two-group comparisons. For behavioral data collected across multiple time points, a two-way repeated-measures analysis of variance (ANOVA) was applied, followed by Tukey’s post-hoc test for pairwise comparisons. For single-time-point multi-group datasets, one-way ANOVA followed by Tukey’s post-hoc test was used for intergroup comparisons. All statistical tests were two-tailed, and a *P* value < 0.05 was considered statistically significant. No outlier testing was conducted in this study. All quantitative data from eligible experimental samples were retained for final statistical analyses, and no data points were excluded post hoc.

## 3 Results

### 3.1 Sodium selenite improves functional recovery and preserves oligodendrocytes following spinal cord injury

To evaluate the therapeutic effects of sodium selenite (SS) following spinal cord injury (SCI), hindlimb locomotor function was assessed using the Basso–Beattie–Bresnahan (BBB) locomotor rating scale. As shown in Figure 1C, rats treated with SS exhibited significantly higher BBB scores than untreated SCI rats at 21 and 42 days post-injury (dpi), indicating improved functional recovery following SS administration. Because the survival of oligodendrocytes is critical for maintaining axonal conduction and neurological function after SCI (Yao et al., 2019; Shi et al., 2020; Ge et al., 2022), we next examined whether SS affected oligodendrocyte preservation within the injured spinal cord. Immunohistochemical analysis revealed a marked decrease in the number of CC1-positive (CC1^+^) mature oligodendrocytes at the lesion epicenter following SCI compared with the Sham group (Figures 1A,B). Notably, SS treatment significantly increased the number of CC1^+^ cells relative to the SCI group, indicating attenuation of oligodendrocyte loss after injury (Figure 1B). To further investigate whether the protective effects of SS were associated with GPX4-dependent transcriptional regulation, actinomycin-D (Act-D) was co-administered with SS. The SS-induced increase in CC1^+^ oligodendrocytes was significantly attenuated by Act-D treatment (Figure 1B), suggesting that the beneficial effects of SS on oligodendrocyte preservation are transcriptionally regulated. Taken together, these findings demonstrate that SS promotes locomotor recovery and preserves mature oligodendrocytes following SCI, supporting a potential link between oligodendrocyte protection and improved neurological function.

**Figure 1:**
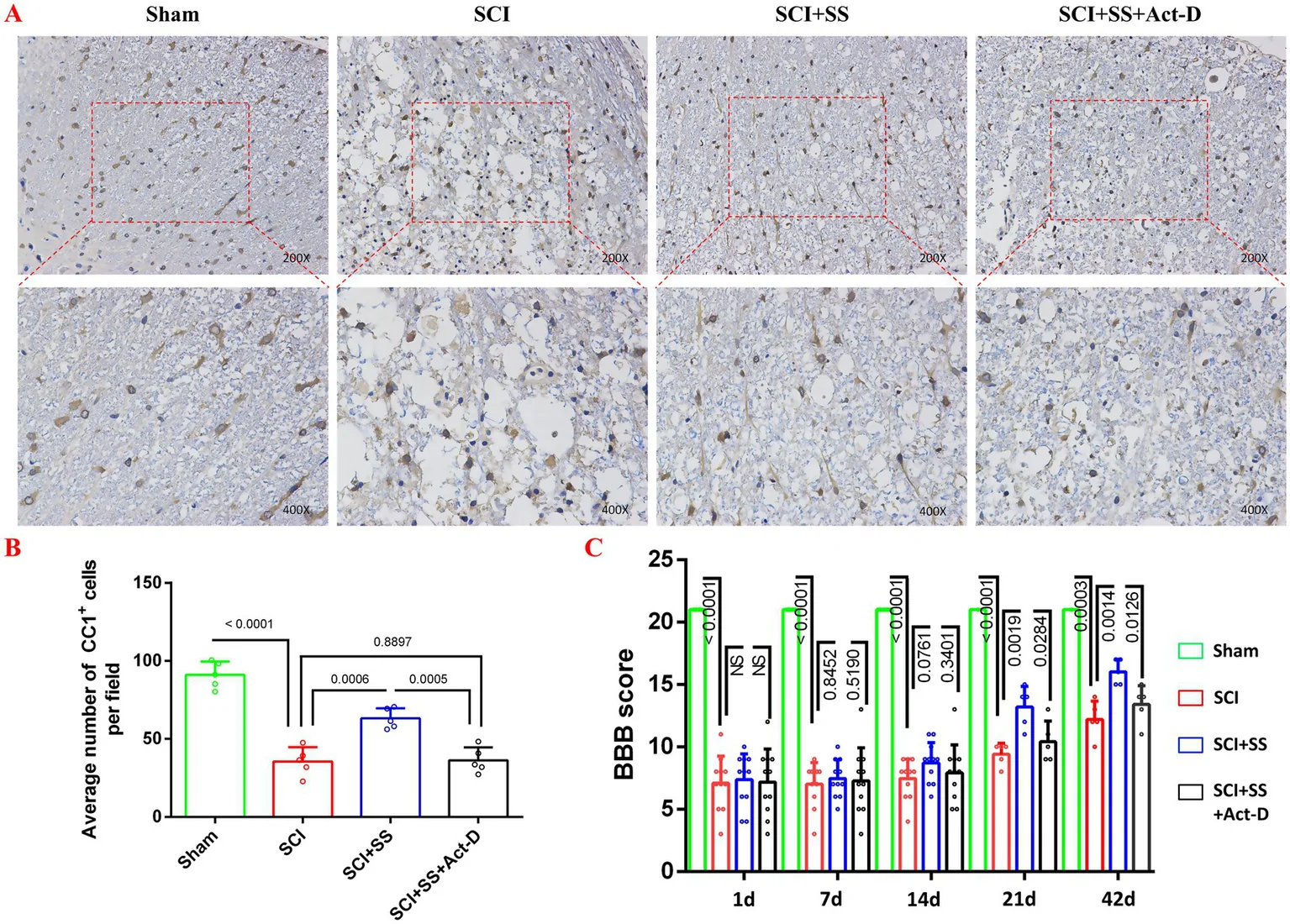
**(A)** Sodium selenite promotes functional recovery following spinal cord injury by mitigating white matter injury. Representative immunohistochemical images showing the presence of CC1^+^ cells in the epicenter of the injured spinal cord in each group at 14 days post-SCI. **(B)** Bar chart quantifying the average number of CC1^+^ cells per field (200×; top row) from panel **(A)**. Data are presented as mean ± SD; *n* = 5 tissue specimens per group. One-way ANOVA showed a significant overall difference among groups (*F*_(3, 16)_ = 52.02, *p* < 0.0001), followed by Tukey’s post-hoc test for pairwise comparisons. **(C)** Bar chart quantifying the post-injury motor function of rats in different groups on days 1, 7, 14, 21 and 42 post-SCI, evaluated by the Basso, Beattie and Bresnahan (BBB) score; two-way repeated-measures ANOVA followed by Tukey’s post-hoc test.

### 3.2 Sodium selenite preserves myelin integrity and attenuates white matter injury following spinal cord injury

To assess white matter preservation and myelin integrity, Luxol Fast Blue (LFB) staining was performed at 14 days post-injury (dpi). LFB staining revealed substantial tissue loss and white matter disruption at the lesion epicenter following SCI, whereas SS treatment markedly preserved spinal cord architecture and white matter structure (Figures 2A,C). These findings suggest that SS alleviates SCI-induced white matter damage. To further evaluate myelin integrity, immunofluorescence staining was performed to examine myelin basic protein (MBP), a major structural component required for the formation and maintenance of compact myelin (Boggs, 2006). As shown in Figures 3A,B, MBP expression was significantly reduced following SCI compared with the Sham group, indicating severe myelin loss. Notably, SS administration significantly restored MBP expression, suggesting enhanced preservation of myelin structure after injury. Because myelin degradation is a hallmark of white matter injury, degraded myelin basic protein (dMBP) expression was subsequently assessed. Immunofluorescence analysis demonstrated a marked increase in dMBP immunoreactivity after SCI, whereas SS treatment significantly reduced dMBP fluorescence intensity (Figures 3C,D), indicating attenuation of myelin degradation. To further validate these observations, western blot analysis was performed to quantify the expression of dMBP and SMI-32, a marker of axonal injury and degeneration. As shown in Figures 2B–E, both dMBP and SMI-32 expression levels were significantly elevated in the injured spinal cord following SCI. SS treatment markedly suppressed the SCI-induced upregulation of both proteins, indicating reduced myelin breakdown and axonal damage. It should be noted that the dMBP signal in the Sham group appeared barely detectable by western blot, whereas dMBP immunoreactivity was clearly present in the Sham group by immunofluorescence staining. This apparent discrepancy is not contradictory but rather reflects differences in detection sensitivity between the two techniques. Because the basal level of dMBP in intact myelin is extremely low, the sham dMBP signal fell below the detection threshold of the western blot system under the present loading conditions (50 μg of protein per lane), whereas immunofluorescence, which provides signal amplification and higher sensitivity for detecting low-abundance antigens in situ, could resolve this low basal expression. Importantly, the two techniques yielded fully consistent results, both demonstrating a marked SCI-induced elevation of dMBP that was significantly attenuated by SS treatment. Taken together, these findings demonstrate that SS preserves myelin integrity, reduces myelin degradation and axonal injury, and ultimately attenuates white matter injury following SCI.

**Figure 2:**
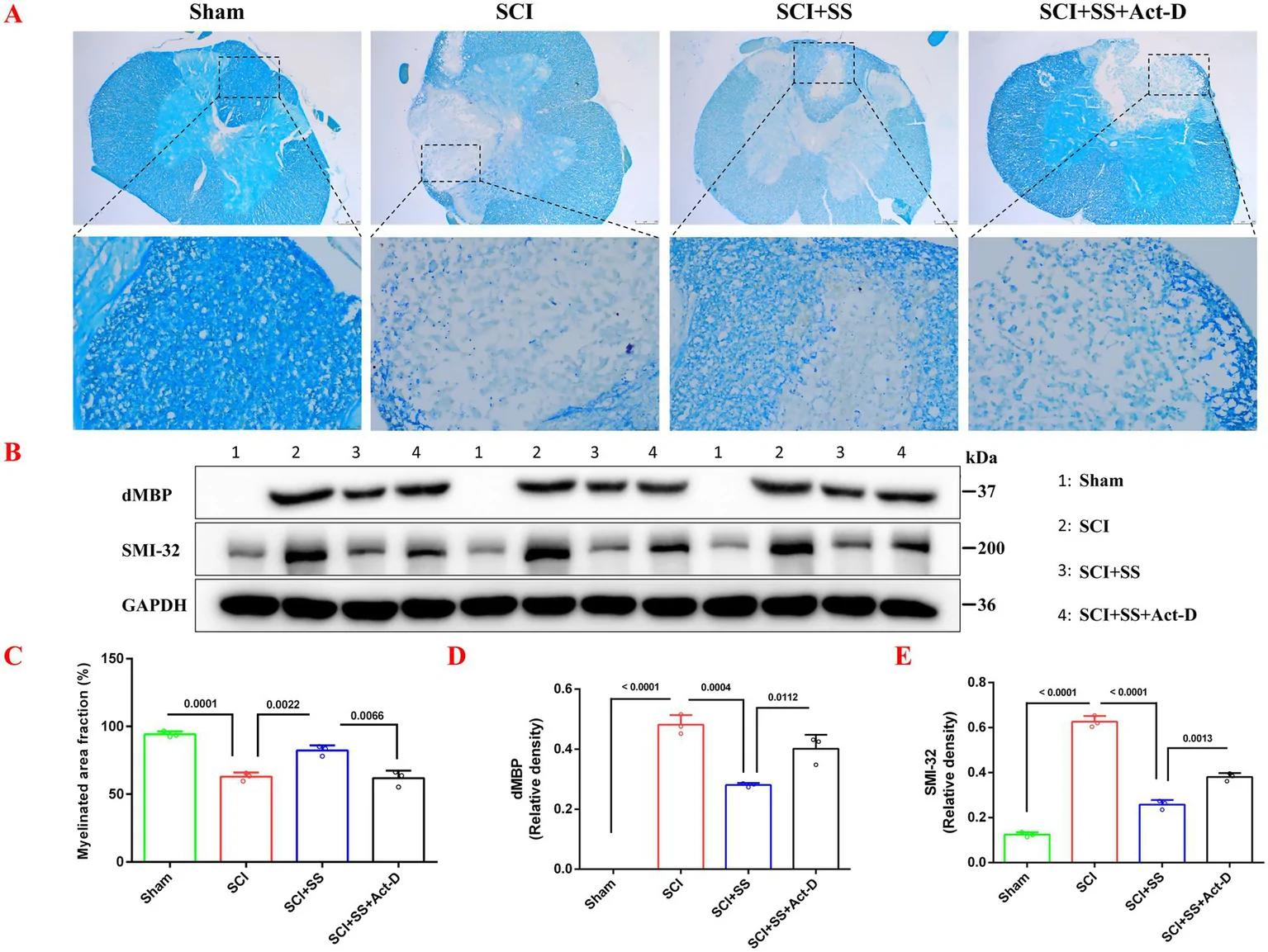
Sodium selenite alleviates white matter injury (WMI), myelin degradation, and axonal injury following spinal cord injury. **(A)** Representative Luxol Fast Blue (LFB) images show the myelin structure of the spinal cord in each group at 14 days post-SCI. Images include low-magnification overviews (top row), with dashed boxes indicating regions of interest that are shown at intermediate magnification (middle row). Scale bar: 250 µm (top row). **(B)** Immunoblot bands representing the expression levels of dMBP and SMI-32 in each group on day 14 post-SCI. GAPDH served as an internal control. **(D, E)** Semi-quantitative analysis of dMBP **(D)** and SMI-32 **(E)** expression levels from panel **(B)**. Data are presented as mean ± SD; *n* = 3 tissue specimens per group. One-way ANOVA showed a significant overall difference among groups (*F*_(3, 8)_ = 164.5, *p* < 0.0001), followed by Tukey’s post-hoc test for pairwise comparisons **(D)**. Data are presented as mean ± SD; *n* = 3 tissue specimens per group. One-way ANOVA showed a significant overall difference among groups (*F*_(3, 8)_ = 369.8, *p* < 0.0001), followed by Tukey’s post-hoc test for pairwise comparisons **(E)**. Scale bars are indicated in each image. **(C)** Bar chart quantifying the myelinated area fraction (%) in each group. Data are presented as mean ± SD; *n* = 3 tissue specimens per group. One-way ANOVA showed a significant overall difference among groups (*F*_(3, 8)_ = 49.10, *p* < 0.0001), followed by Tukey’s post-hoc test for pairwise comparisons.

**Figure 3:**
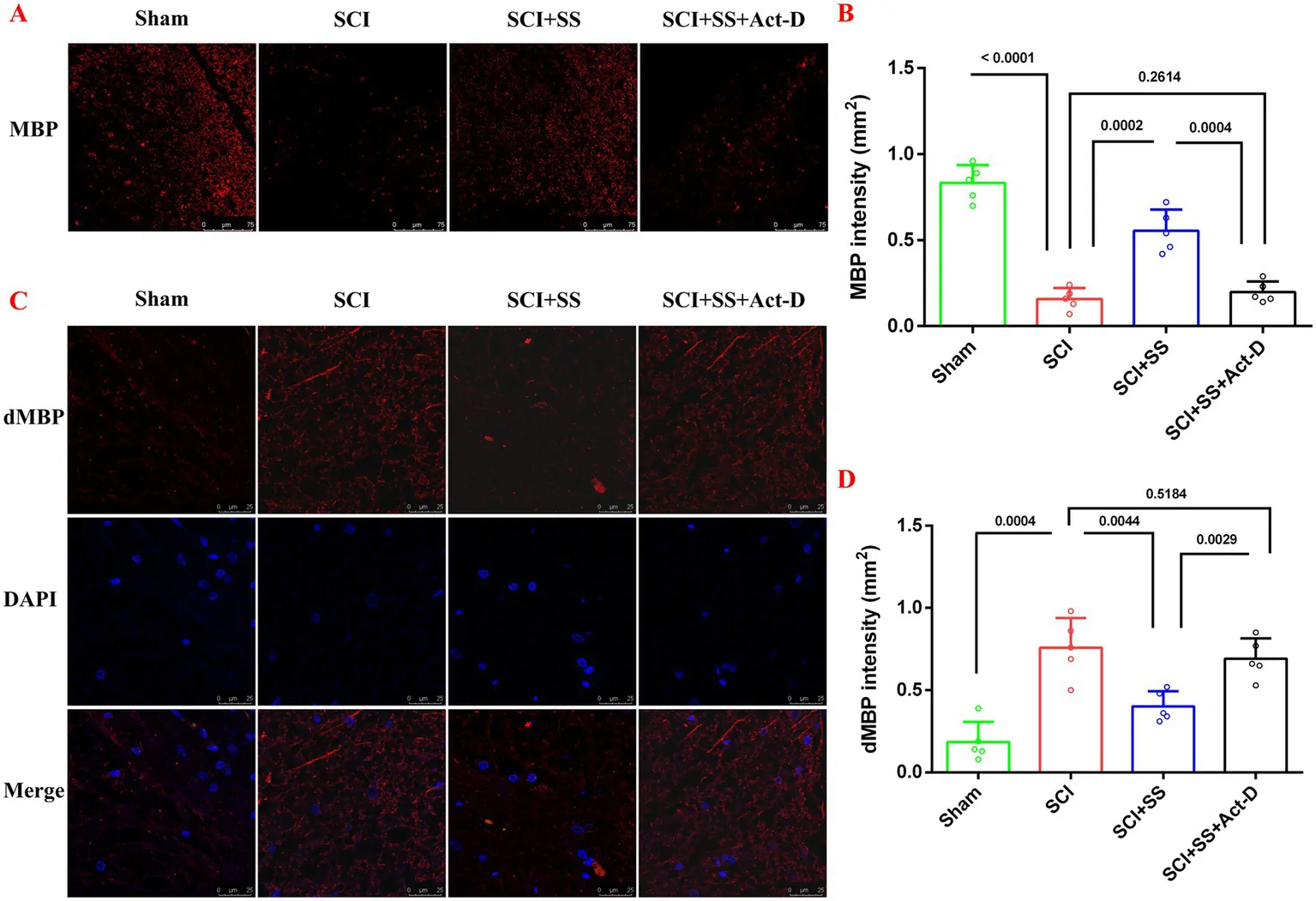
Sodium selenite alleviates demyelination and promotes myelin repair after spinal cord injury. **(A)** Immunostaining images showing MBP expression in each group at 14 days post-SCI. Scale bar: 75 μm. **(B)** Semi-quantitative bar chart quantifying the optical density of MBP across groups. Data are presented as mean ± SD; *n* = 5 tissue specimens per group. One-way ANOVA showed a significant overall difference among groups (*F*_(3, 16)_ = 60.47, *p* < 0.0001), followed by Tukey’s post-hoc test for pairwise comparisons. **(C)** Immunostaining images showing dMBP expression in each group at 14 days post-SCI, with nuclei counterstained with DAPI. Scale bar: 25 μm. **(D)** Semi-quantitative bar chart quantifying the optical density of dMBP across groups. Data are presented as mean ± SD; *n* = 5 tissue specimens per group. One-way ANOVA showed a significant overall difference among groups (*F*_(3, 16)_ = 19.90, *p* < 0.0001), followed by Tukey’s post-hoc test for pairwise comparisons.

### 3.3 Sodium selenite attenuates ferroptosis-associated demyelination following spinal cord injury

Given that oligodendrocytes are highly susceptible to ferroptotic cell death and are essential for myelin maintenance, we next investigated whether the protective effects of sodium selenite (SS) on white matter integrity were associated with suppression of ferroptosis. To further evaluate myelin preservation, myelin sheath ultrastructure was examined by transmission electron microscopy (TEM). As shown in Figures 4A,B, SCI resulted in a marked reduction in myelin sheath thickness compared with the Sham group, indicating substantial demyelination. In contrast, SS treatment significantly preserved myelin sheath thickness, suggesting attenuation of myelin damage following injury. Consistent with these ultrastructural observations, western blot analysis demonstrated that the expression of degraded myelin basic protein (dMBP), a marker of myelin breakdown, was significantly increased after SCI and was markedly reduced by SS treatment (Figures 2B,D). Similar results were obtained by immunofluorescence staining, which showed that SS significantly decreased dMBP immunoreactivity within the injured spinal cord (Figures 3C,D). To determine whether ferroptosis contributed to SCI-induced demyelination, mitochondrial morphology was assessed by TEM. Ultrastructural analysis revealed a significant increase in the proportion of shrunken mitochondria with condensed membrane density, a characteristic morphological feature of ferroptosis, in the SCI group. Notably, SS treatment markedly reduced the proportion of these ferroptotic mitochondria (Figures 4C,D). These mitochondrial images were specifically obtained from oligodendrocytes within the white matter of the lesion epicenter. To further validate the involvement of ferroptosis, we examined the expression of iron regulatory protein 2 (IRP2), a ferroptosis-associated protein involved in cellular iron metabolism. Western blot analysis demonstrated that IRP2 expression was significantly elevated following SCI and was substantially reduced after SS administration (Figures 5B,D). The concordance between mitochondrial ultrastructural changes and IRP2 expression further supports the anti-ferroptotic effect of SS in the injured spinal cord. Taken together, these findings suggest that ferroptosis contributes to SCI-induced demyelination and that SS alleviates white matter injury, at least in part, through suppression of ferroptosis.

**Figure 4:**
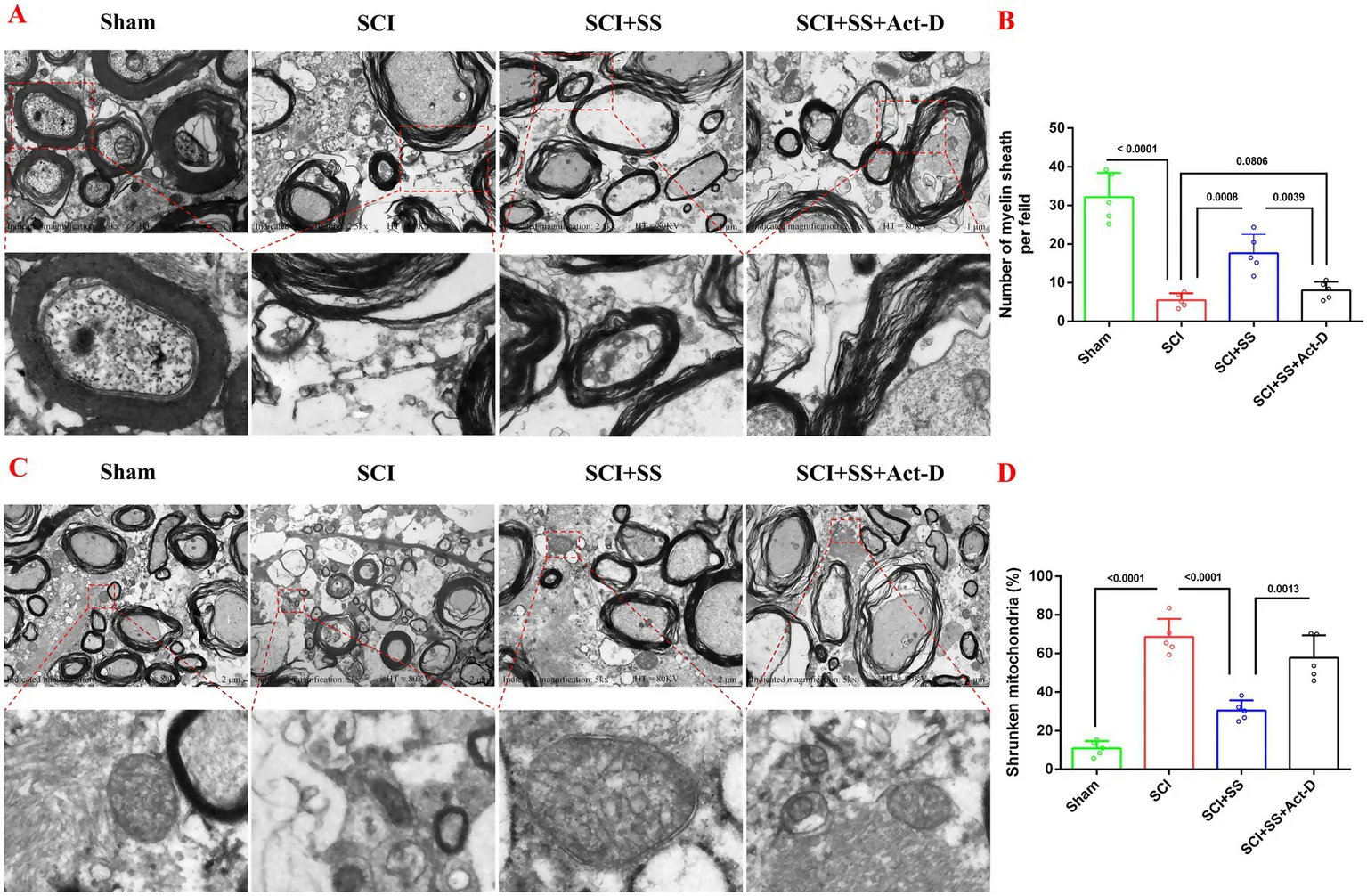
Sodium selenite reduces demyelination by mitigating ferroptosis following spinal cord injury. All TEM images were acquired from oligodendrocytes within the white matter of the spinal cord epicenter. **(A)** Representative TEM images of myelin sheaths at the spinal cord epicenter 14 days post-SCI. Scale bar: 1 μm (top row). **(B)** Quantitative analysis of the number of myelin sheaths per field corresponding to panel **(A)**. Data are presented as mean ± SD; *n = 5 tissue specimens per group*. One-way ANOVA showed a significant overall difference among groups (*F*_(3, 16)_ = 40.39, *p* < 0.0001), followed by Tukey’s post-hoc test for pairwise comparisons. **(C)** Representative TEM images showing morphological changes of mitochondria in each group at 14 days post-SCI. Scale bars: 2 μm (top row). **(D)** Quantitative analysis of the percentage of shrunken mitochondria (a hallmark of ferroptosis) corresponding to panel **(C)**. Data are presented as mean ± SD; *n* = 5 tissue specimens per group. One-way ANOVA showed a significant overall difference among groups (*F*_(3, 16)_ = 52.16, *p* < 0.0001), followed by Tukey’s post-hoc test for pairwise comparisons.

**Figure 5:**
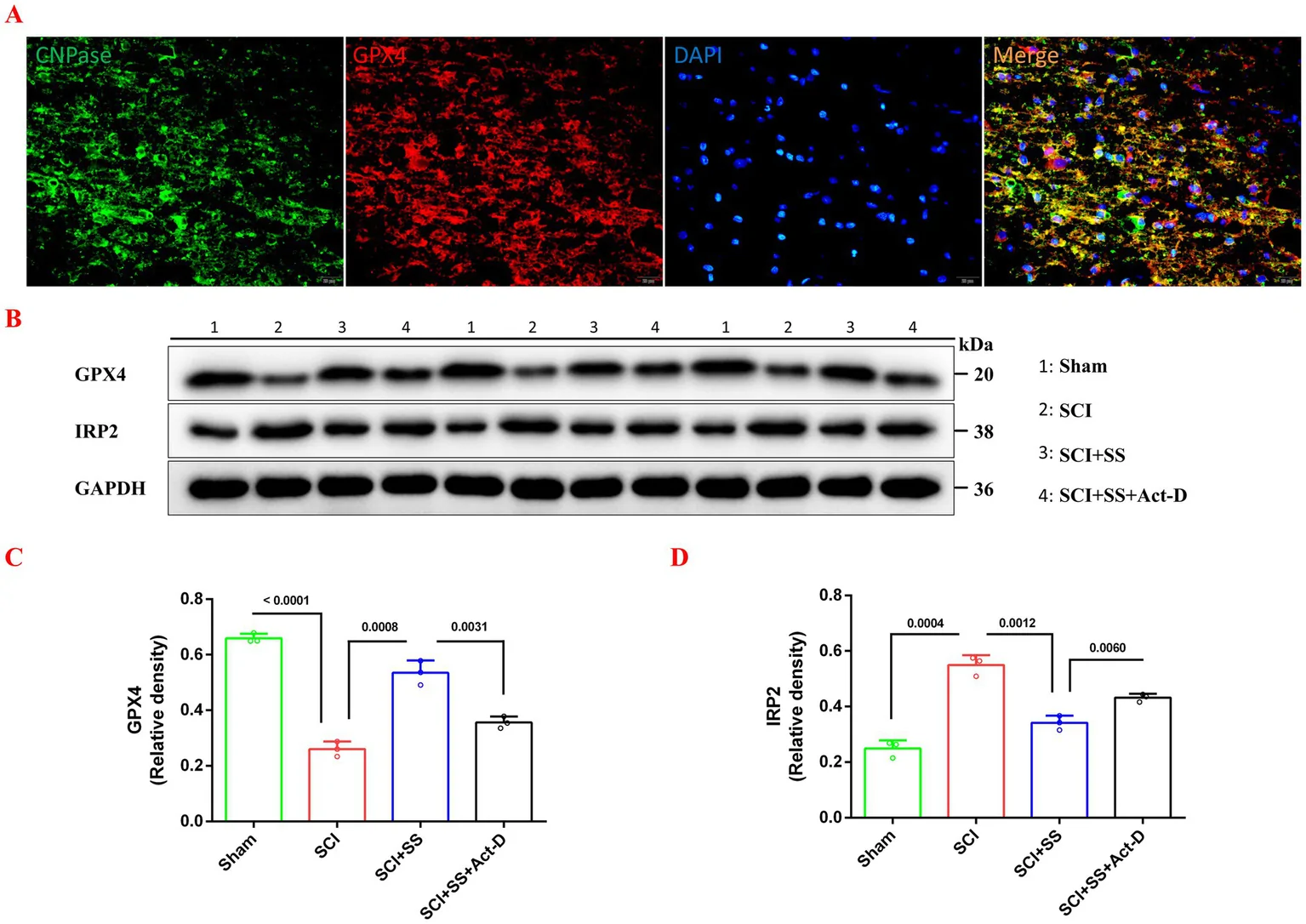
Sodium selenite mitigates ferroptosis by upregulating GPX4 expression following spinal cord injury (SCI). **(A)** Representative immunostaining images of CNPase (green) and GPX4 (red) in the injured rat spinal cord. Nuclei were counterstained with DAPI. Scale bar: 20 μm. *n* = 3 tissue specimens per group. **(B)** Immunoblot bands representing the expression levels of GPX4 and IRP2 in each group at 14 days post-SCI. GAPDH served as an internal control. **(C, D)** Semi-quantitative analysis of the expression levels of GPX4 **(C)** and IRP2 **(D)** from panel **(B)**. Data are presented as mean ± SD; *n *= 3 tissue specimens per group. One-way ANOVA showed a significant overall difference among groups (*F*_(3, 8)_ = 113.4, *p* < 0.0001), followed by Tukey’s post-hoc test for pairwise comparisons **(C)**. Data are presented as mean ± SD; *n *= 3 tissue specimens per group. One-way ANOVA showed a significant overall difference among groups (*F*_(3, 8)_ = 65.72, *p* < 0.0001), followed by Tukey’s post-hoc test for pairwise comparisons **(D)**.

### 3.4 Sodium selenite inhibits oligodendrocyte ferroptosis through upregulation of GPX4 following spinal cord injury

The results indicate that SS effectively reduces oligodendrocyte precursor cell (OPC) ferroptosis, primarily by upregulating GPX4 expression. To elucidate the underlying mechanism, we first conducted immunostaining to visualize CNPase (green) and GPX4 (red) in rat spinal cords following injury (Figure 5A). Immunofluorescence staining further demonstrated that SS administration significantly enhanced GPX4 expression (Figures 6A,B). Additionally, immunoblot analysis revealed a marked downregulation of GPX4 protein expression post-spinal cord injury, which was notably reversed by SS treatment (Figures 5B,C), and an upregulation of IRP2 protein expression post-spinal cord injury, which was notably reversed by SS treatment (Figures 5B,D). To further investigate the role of SS in attenuating ferroptosis, our results showed that SS not only upregulated GPX4 expression but also reduced the number of shrunken mitochondria; however, this GPX4 upregulation was significantly abolished by actinomycin D. Collectively, these findings suggest that SS inhibits OPC ferroptosis through the upregulation of GPX4 expression.

**Figure 6:**
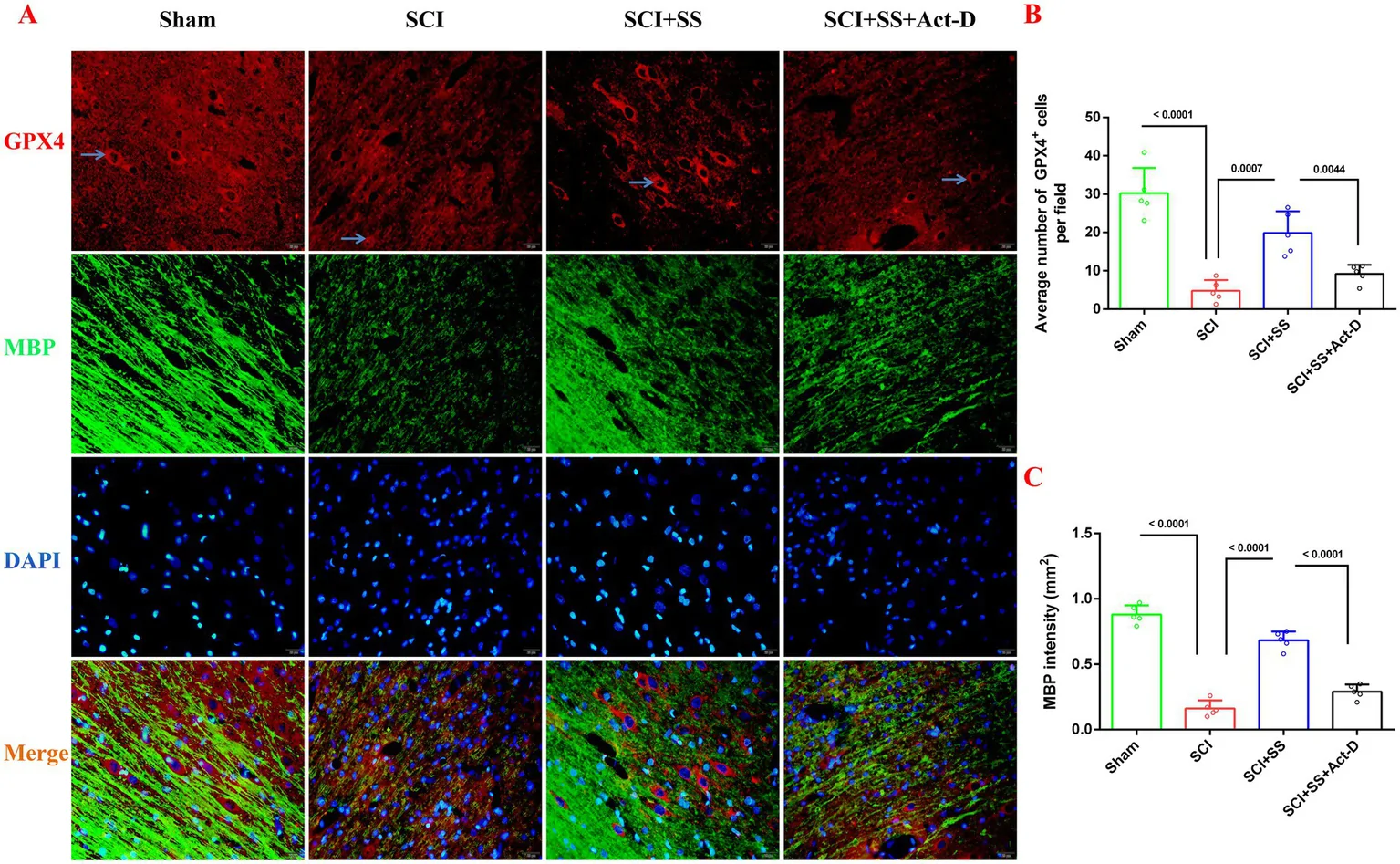
Sodium selenite reduces demyelination by upregulating GPX4 expression following spinal cord injury. **(A)** Representative immunostaining images showing the expression of GPX4^+^ (red) cells and MBP (green) in the epicenter of the injured spinal cord of each group at 14 days post-SCI. Nuclei were counterstained with DAPI. Scale bar: 20 μm. **(B)** Bar chart quantifying the average number of GPX4^+^ cells per field from panel A. Data are presented as mean ± SD; *n* = 5 tissue specimens per group. One-way ANOVA showed a significant overall difference among groups (*F*_(3, 16)_ = 29.18, *p *< 0.0001), followed by Tukey’s post-hoc test for pairwise comparisons. **(C)** Semi-quantitative bar chart quantifying the optical density of MBP for each group. Data are presented as mean ± SD; *n* = 5 tissue specimens per group. One-way ANOVA showed a significant overall difference among groups (*F*_(3, 16)_ = 138.7, *p* < 0.0001), followed by Tukey’s post-hoc test for pairwise comparisons.

## 4 Discussion

Spinal cord injury (SCI) frequently results in persistent motor, sensory, and autonomic dysfunction, and accumulating evidence indicates that the extent of white matter preservation is a major determinant of neurological recovery following injury (Shi et al., 2023; Ahuja et al., 2017). Because oligodendrocytes are responsible for the formation and maintenance of myelin sheaths, their loss contributes directly to demyelination, axonal dysfunction, and progressive white matter injury (WMI). Recent studies have identified ferroptosis, an iron-dependent form of regulated cell death driven by lipid peroxidation, as an important contributor to secondary injury after SCI (Shi et al., 2023; Yao et al., 2019; Ge et al., 2022) and to oligodendrocyte loss in demyelinating conditions more broadly (Jhelum et al., 2020). Notably, oligodendrocytes contain abundant polyunsaturated fatty acids, making them particularly vulnerable to ferroptotic damage (Simons and Nave, 2015), which can compromise myelin integrity and axonal conduction, thereby exacerbating neurological dysfunction (Simons and Nave, 2015). In the present study, we provide evidence that SCI is associated with a marked reduction in glutathione peroxidase 4 (GPX4) expression at the lesion epicenter, accompanied by increased ferroptotic injury, oligodendrocyte loss, demyelination, and neurological impairment. We further demonstrate that sodium selenite (SS) treatment significantly restored GPX4 expression, preserved oligodendrocyte survival, reduced ferroptosis-associated mitochondrial damage and iron regulatory protein 2 (IRP2) expression, attenuated white matter injury, and improved locomotor recovery after SCI. Importantly, the beneficial effects of SS were largely abolished by co-administration of actinomycin D (Act-D), indicating that transcriptional regulation of GPX4 is a key mechanism underlying the neuroprotective actions of SS (Figure 7). Collectively, these findings support a model in which SS enhances GPX4-mediated ferroptosis resistance in oligodendrocytes, thereby preserving white matter integrity and promoting functional recovery following SCI.

**Figure 7:**
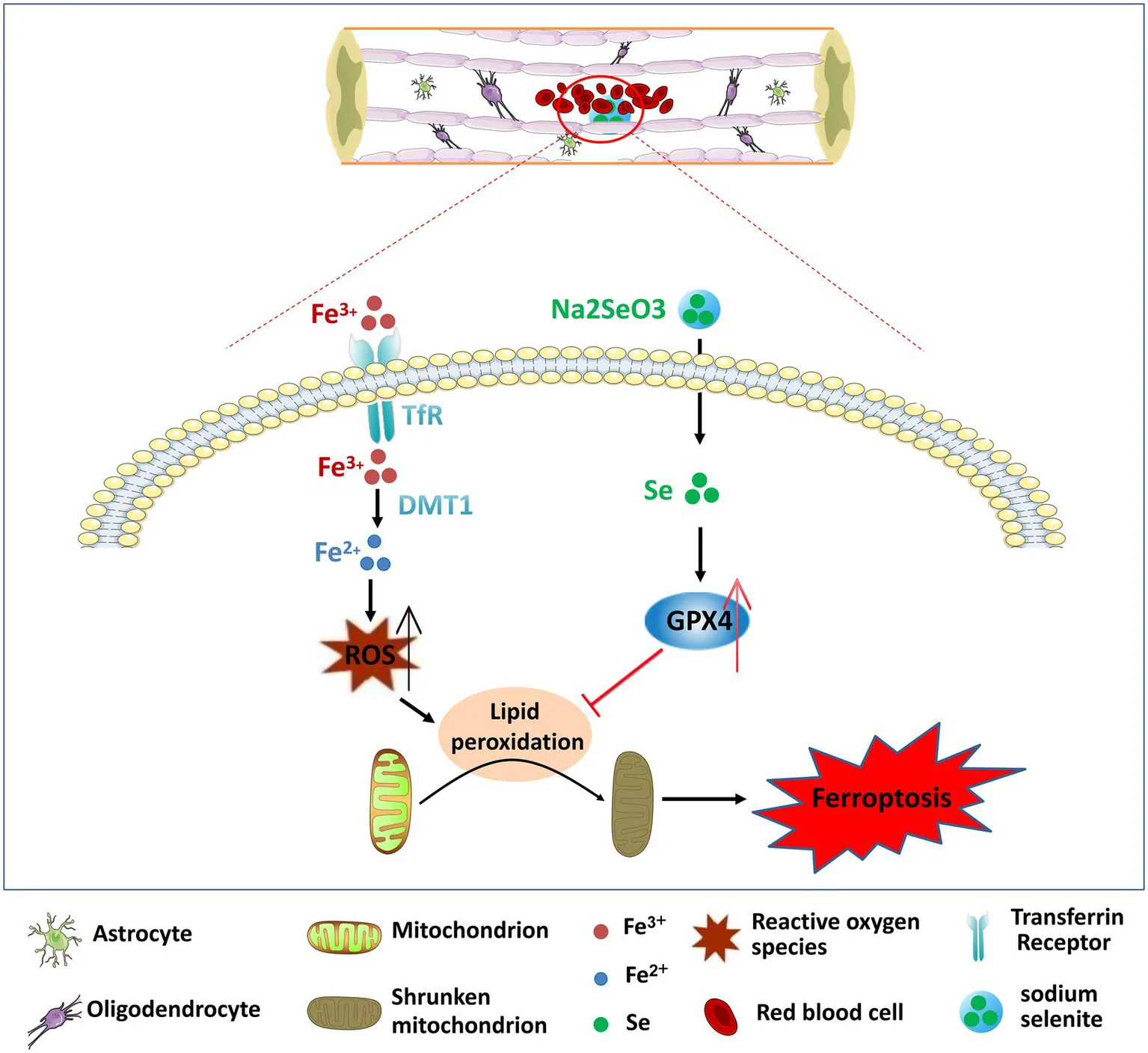
Schematic illustration of the potential therapeutic effect of sodium selenite on ferroptosis in SCI and its underlying mechanism.

The apparent dissociation between structural preservation and functional recovery deserves further discussion. At 14 days post-SCI, LFB staining revealed a significant difference in white matter preservation between the SCI + SS and SCI + SS + Act-D groups, whereas the BBB scores of these two groups did not differ significantly at the same time point. We believe that this discrepancy reflects the different biological information captured by the two outcome measures rather than a contradiction in the data. First, LFB staining directly quantifies myelin preservation at the lesion epicenter, whereas the BBB score is an integrative behavioral readout that reflects the net functional output of multiple descending and ascending pathways. Functional recovery generally lags behind structural preservation after SCI, and the structural benefits conferred by SS at 14 days post-injury may not yet have translated into measurable locomotor gains, which became statistically significant only at 21 and 42 days post-injury (Figure 1C). Second, the BBB scale is an ordinal, stepwise scoring system with limited resolution at the intermediate recovery phase, and inter-animal variability in locomotor performance further reduces its discriminative power at a single time point (Basso et al., 1995). Third, a critical threshold of spared white matter, particularly within the ventrolateral funiculus, is required before structural preservation is translated into detectable locomotor recovery (Fehlings and Tator, 1995; Schucht et al., 2002). It is therefore plausible that, at 14 days post-SCI, the difference in myelin preservation between the SCI + SS and SCI + SS + Act-D groups had not yet surpassed this functional threshold, whereas the cumulative white matter protection achieved by SS over subsequent weeks led to the significant BBB differences observed at 21 and 42 days post-injury. Taken together, these findings indicate that structural preservation is a prerequisite for, but not equivalent to, functional recovery, and that the early structural benefits of SS are followed by delayed, yet significant, functional gains.

As a central regulator of ferroptosis, GPX4 plays a critical role in maintaining cellular redox homeostasis by reducing toxic lipid hydroperoxides to their corresponding lipid alcohols, thereby preventing the propagation of lipid peroxidation and subsequent ferroptotic cell death (Yang et al., 2014; Dixon and Stockwell, 2019). Consequently, loss or inactivation of GPX4 compromises membrane integrity and renders cells highly susceptible to ferroptosis (Dixon and Stockwell, 2019). Given their high lipid content and enrichment in polyunsaturated fatty acids, oligodendrocytes are particularly vulnerable to lipid peroxidation-mediated injury, suggesting that strategies aimed at preserving or enhancing GPX4 activity may be especially effective for limiting white matter injury following SCI. Consistent with previous reports demonstrating reduced GPX4 expression after SCI (Yao et al., 2019), our findings confirmed a marked decrease in GPX4 levels within the injured spinal cord. Importantly, we further demonstrated that sodium selenite (SS) treatment significantly restored GPX4 expression, accompanied by reduced ferroptosis-associated pathological changes, including decreased IRP2 expression and attenuation of ferroptotic mitochondrial morphology. It is important to clarify how IRP2 relates to the function and activity of GPX4. IRP2 is a master post-transcriptional regulator of cellular iron metabolism that stabilizes transferrin receptor 1 (TfR1) mRNA and represses ferritin translation, thereby expanding the labile iron pool that fuels Fenton reaction-driven lipid peroxidation (Cardona and Montgomery, 2023). Excessive iron-catalyzed lipid peroxidation consumes glutathione and overwhelms the reducing capacity of GPX4, compromising GPX4-mediated detoxification of phospholipid hydroperoxides and thereby promoting ferroptosis; conversely, IRP2 knockdown or functional inhibition has been shown to reduce ferroptosis sensitivity, an effect demonstrated in hepatic stellate cells (Li et al., 2020). Accordingly, the SCI-induced upregulation of IRP2 and downregulation of GPX4 observed in the present study are functionally linked: iron overload increases the peroxidative burden that GPX4 must detoxify, and the SS-induced reduction of IRP2 together with restoration of GPX4 indicates amelioration of this iron-driven peroxidative stress, ultimately suppressing oligodendrocyte ferroptosis. These observations suggest that GPX4 upregulation contributes to the anti-ferroptotic effects of SS following SCI. Furthermore, double immunofluorescence staining for CNPase and GPX4 revealed that GPX4 was predominantly expressed in oligodendrocytes and that SS treatment increased the abundance of GPX4-positive oligodendrocytes within the injured spinal cord. Together with the observed preservation of myelin integrity and reduction in demyelination, these findings support the notion that SS-mediated GPX4 upregulation enhances oligodendrocyte resistance to ferroptotic injury, thereby contributing to white matter preservation and improved neurological recovery after SCI.

Selenium supplementation has previously been investigated as a potential therapeutic strategy for SCI, with most studies attributing its neuroprotective effects to attenuation of oxidative stress and inhibition of reactive oxygen species (ROS)-mediated apoptotic pathways (Yeo et al., 2008), as well as to immunomodulatory effects (Garcia et al., 2022). In contrast, the present study expands the current understanding of selenium-mediated neuroprotection by identifying suppression of ferroptosis as an additional and potentially important mechanism through which sodium selenite (SS) preserves white matter integrity and promotes functional recovery after SCI. These findings suggest that modulation of ferroptosis, particularly through the GPX4 pathway, may represent a more specific therapeutic target for limiting secondary injury following SCI. Emerging evidence indicates that susceptibility to ferroptosis and the mechanisms governing ferroptosis resistance vary among different cell types. GPX4 appears to be particularly important in cells that rely heavily on glutathione (GSH)-dependent antioxidant defenses, including neurons, oligodendrocytes, and renal tubular epithelial cells (Seibt et al., 2019; Kim et al., 2021). In these cell populations, impairment of GPX4 activity readily promotes lipid peroxidation and ferroptotic cell death. By contrast, other cell types may exhibit greater dependence on alternative ferroptosis-defense systems, such as the ferroptosis suppressor protein 1 (FSP1)/coenzyme Q10 (CoQ_10_) pathway, which suppresses lipid peroxidation independently of GPX4 (Bersuker et al., 2019; Doll et al., 2019). The preferential localization of GPX4 within oligodendrocytes observed in the present study, together with the robust protective effects of SS on GPX4 expression and oligodendrocyte survival, supports the notion that the GPX4-dependent ferroptosis defense pathway is particularly relevant to white matter preservation after SCI.

Although the present study demonstrates that sodium selenite (SS) treatment is associated with increased GPX4 expression following SCI, the precise molecular mechanisms responsible for this regulation remain to be fully elucidated. Notably, the observation that actinomycin D (Act-D) largely abolished the protective effects of SS suggests that GPX4 upregulation occurs, at least in part, through transcriptional regulation. Act-D is not a classical ferroptosis inducer; however, recent studies have shown that, in cancer cell models, Act-D can markedly enhance ferroptosis sensitivity through multiple pathways, including downregulation of the cystine/glutamate antiporter system Xc− (SLC7A11) with secondary inhibition of glutathione synthesis, induction of ferritinophagy, and consequent inhibition of GPX4 expression (Xue et al., 2024). In the present study, Act-D was used specifically as a transcriptional inhibitor to determine whether SS-induced GPX4 upregulation is required for the neuroprotective effects of SS, rather than as a ferroptosis inducer. We acknowledge that an Act-D-alone treatment group was not included in the present experimental design, so potential direct effects of Act-D on ferroptosis sensitivity cannot be entirely excluded. Nevertheless, the complete abrogation of SS-conferred oligodendrocyte protection, white matter preservation, and functional recovery by Act-D strongly supports the interpretation that the therapeutic effects of SS are mediated, at least in part, through the transcriptional upregulation of GPX4. However, the specific transcription factors and signaling pathways involved were not directly examined in the current study. Our findings are consistent with previous work in a murine model of intracerebral hemorrhage (ICH), which demonstrated that selenium promotes GPX4 expression by activating the transcription factors AP-2 gamma (TFAP2C) and specificity protein 1 (Sp1), thereby enhancing resistance to ferroptosis (Alim et al., 2019). Based on these observations, it is plausible that a similar transcriptional mechanism may operate in the injured spinal cord. Specifically, SS treatment may enhance the activity or expression of Sp1, TFAP2C, or other transcriptional regulators that control GPX4 gene expression, ultimately increasing cellular resistance to ferroptotic injury. Nevertheless, this hypothesis remains speculative and requires direct experimental validation. Future studies incorporating chromatin immunoprecipitation assays, promoter activity analyses, and targeted manipulation of candidate transcription factors will be necessary to determine whether Sp1, TFAP2C, or related regulatory pathways mediate selenium-induced GPX4 upregulation following SCI.

Secondary injury following SCI is a highly dynamic and multifactorial process involving extensive interactions among multiple cell types, signaling pathways, and intracellular organelles (Shi et al., 2023; Ahuja et al., 2017). This pathological cascade is generally characterized by several interconnected processes, including oxidative stress, metabolic dysfunction, neuroinflammation, and multiple forms of regulated cell death. Among these, oxidative stress is considered a major upstream event that links excitotoxicity, inflammation, and cell death through excessive generation of reactive oxygen species (ROS). Consistent with this concept, our previous studies demonstrated substantial ROS accumulation after SCI, which contributes to ferroptotic injury of oligodendrocytes (Shi et al., 2020; Shi et al., 2023). Neuroinflammation represents another critical component of secondary SCI pathology. Oxidative stress can stimulate the production of pro-inflammatory mediators, whereas preservation of mitochondrial function and cellular homeostasis may limit amplification of inflammatory responses. Importantly, ferroptotic death of oligodendrocytes and subsequent demyelination generate large amounts of myelin debris within the injured spinal cord. Inefficient clearance of this debris by macrophages and microglia can disrupt lipid metabolism and promote a persistent pro-inflammatory microenvironment, which in turn contributes to astrocyte activation and glial scar formation. This self-perpetuating cycle of demyelination and inflammation may impair oligodendrocyte precursor cell differentiation and remyelination, thereby exacerbating long-term neurological dysfunction. Supporting this concept, our previous work demonstrated that inhibition of oligodendrocyte ferroptosis attenuates abnormal activation of microglia and astrocytes following SCI, suggesting a close relationship between ferroptotic white matter injury and neuroinflammatory responses (Ge et al., 2022). Although inflammatory markers were not directly examined in the present study, the observed reduction in oligodendrocyte loss, demyelination, and ferroptosis following sodium selenite (SS) treatment raises the possibility that SS may also indirectly modulate the inflammatory microenvironment after SCI. Taken together, these findings support a model in which oxidative stress promotes GPX4 depletion and ferroptotic injury of oligodendrocytes, leading to demyelination, white matter degeneration, and secondary neuroinflammation. By restoring GPX4 expression and suppressing ferroptosis, SS interrupts this pathological cascade and thereby contributes to white matter preservation and functional recovery following SCI.

Several limitations of the present study should be acknowledged. First, the therapeutic effects of sodium selenite (SS) were evaluated exclusively in a male rat model of contusive SCI. Although this model recapitulates many pathological features of human SCI, the translational potential of SS will require further validation in additional preclinical models, including large-animal or non-rodent models, as well as human cell–based systems, to determine its efficacy across diverse injury paradigms and biological contexts. Second, while our findings identify GPX4-mediated ferroptosis inhibition as a key mechanism underlying the protective effects of SS, we did not investigate potential interactions between ferroptosis and other forms of regulated cell death, such as apoptosis, necroptosis, or pyroptosis. Given the complex pathophysiology of secondary SCI, these pathways may interact with ferroptosis and collectively contribute to oligodendrocyte loss and white matter degeneration. In addition, neuroinflammatory responses, including microglial activation, macrophage polarization, and reactive astrogliosis, were not directly assessed and may represent important downstream targets influenced by SS treatment. Third, selenium possesses a relatively narrow therapeutic window, and excessive selenium exposure has been associated with adverse biological effects, including increased oxidative stress and systemic toxicity (Rayman, 2012). Therefore, further studies are needed to define the optimal dosage, administration regimen, and therapeutic window for SS in SCI, and additional research is required to validate the findings in female subjects. Advanced delivery approaches, such as biomaterial-based sustained-release systems or targeted nanoparticle carriers, may improve bioavailability while minimizing potential toxicity. Finally, although the present study focused on GPX4 as the principal mediator of SS-induced ferroptosis resistance, other endogenous ferroptosis-suppressive pathways were not examined. Emerging evidence suggests that ferroptosis can also be regulated by GPX4-independent mechanisms, including the ferroptosis suppressor protein 1 (FSP1)-coenzyme Q10 pathway and the GTP cyclohydrolase 1 (GCH1)-tetrahydrobiopterin (BH4) pathway. Whether these signaling networks contribute to the neuroprotective effects of SS following SCI remains unknown and warrants further investigation.

Despite these limitations, the present study provides compelling preclinical evidence supporting sodium selenite (SS) as a potential therapeutic candidate for SCI. As a relatively inexpensive and clinically accessible selenium compound with a well-characterized safety profile at appropriate doses, SS may represent a practical strategy for targeting ferroptosis-mediated white matter injury following SCI. Future studies should focus on several key areas. First, the development of targeted delivery systems, such as biomaterial-based carriers, injectable hydrogels, or nanoparticle-mediated approaches, may improve SS accumulation at the lesion site while minimizing systemic exposure and potential toxicity. Second, the efficacy of SS should be evaluated in chronic SCI models and at extended post-injury time points beyond the 42-day observation period used in the present study to determine whether its protective effects are sustained during long-term tissue remodeling and neurological recovery. Third, given the multifactorial nature of secondary SCI pathology, it will be important to investigate SS in combination with complementary therapeutic strategies, including anti-inflammatory interventions, neuroprotective agents, and axon regeneration-promoting therapies, to determine whether synergistic effects can be achieved. Fourth, further studies are warranted to evaluate whether GPX4 expression or other ferroptosis-related biomarkers may predict treatment responsiveness, thereby facilitating the development of more personalized therapeutic approaches. Beyond SCI, increasing evidence implicates ferroptosis in the pathogenesis of a broad spectrum of central nervous system (CNS) disorders. Therefore, it will be of considerable interest to determine whether the GPX4-dependent neuroprotective effects of SS observed in the present study can be extended to other ferroptosis-associated neurological conditions, including traumatic brain injury, ischemic stroke, and demyelinating diseases such as multiple sclerosis. Such investigations may further expand the translational significance of targeting the selenium–GPX4–ferroptosis axis in neurological disease.

## 5 Conclusion

In conclusion, the present study demonstrates that sodium selenite (SS) treatment significantly upregulates GPX4 expression and suppresses ferroptosis at the spinal cord lesion site following SCI. Through restoration of the GPX4-mediated antioxidant defense pathway, SS promotes oligodendrocyte survival, preserves myelin integrity, and attenuates white matter injury, ultimately leading to improved locomotor recovery in SCI rats. Importantly, the loss of these protective effects following actinomycin D administration further supports a critical role for GPX4 upregulation in mediating the therapeutic actions of SS. Collectively, these findings identify the GPX4–ferroptosis axis as a key therapeutic target for white matter preservation after SCI and highlight SS as a promising pharmacological strategy for mitigating secondary injury and enhancing neurological recovery.

## Data Availability

The original contributions presented in the study are included in the article/supplementary material, further inquiries can be directed to the corresponding author/s.

## References

[ref1] Ahuja C. S. Nori S. Tetreault L. Wilson J. Kwon B. Harrop J. . (2017). Traumatic spinal cord injury—repair and regeneration. Neurosurgery 80, S9–S22. doi: 10.1093/neuros/nyw080, 28350947.

[ref2] Alim I. Caulfield J. T. Chen Y. Swarup V. Geschwind D. H. Ivanova E. . (2019). Selenium drives a transcriptional adaptive program to block ferroptosis and treat stroke. Cell 177, 1262–1279.e25. doi: 10.1016/j.cell.2019.03.032, 31056284.

[ref3] Basso D. M. Beattie M. S. Bresnahan J. C. (1995). A sensitive and reliable locomotor rating scale for open field testing in rats. J. Neurotrauma 12, 1–21. doi: 10.1089/neu.1995.12.1, 7783230.

[ref4] Bersuker K. Hendricks J. M. Li Z. Magtanong L. Ford B. Tang P. H. . (2019). The CoQ oxidoreductase FSP1 acts parallel to GPX4 to inhibit ferroptosis. Nature 575, 688–692. doi: 10.1038/s41586-019-1705-2, 31634900. PMC6883167

[ref5] Bin J. M. Harris S. N. Kennedy T. E. (2016). The oligodendrocyte-specific antibody "CC1" binds Quaking 7. J. Neurochem. 139, 181–186. doi: 10.1111/jnc.13745, 27454326.

[ref6] Boggs J. M. (2006). Myelin basic protein: a multifunctional protein. Cell Mol. Life Sci. 63, 1945–1961. doi: 10.1007/s00018-006-6094-7, 16794783. PMC11136439

[ref7] Cardona C. J. Montgomery M. R. (2023). Iron regulatory proteins: players or pawns in ferroptosis and cancer? Front. Mol. Biosci. 10:1229710. doi: 10.3389/fmolb.2023.1229710, 37457833. PMC10340119

[ref8] Chen X. Comish P. B. Tang D. L. Kang R. (2021). Characteristics and biomarkers of ferroptosis. Front. Cell Dev. Biol. 9:637162. doi: 10.3389/fcell.2021.637162, 33553189. PMC7859349

[ref9] Chen Y. Liu S. Li J. Li Z. Quan J. Liu X. . (2020). The latest view on the mechanism of ferroptosis and its research progress in spinal cord injury. Oxid. Med. Cell. Longev. 2020:6375938. doi: 10.1155/2020/6375938 PMC7474794 32908634

[ref10] Conrad M. Friedmann Angeli J. P. (2015). Glutathione peroxidase 4 (Gpx4) and ferroptosis: what’s so special about it? Mol. Cell. Oncol. 2:e995047. doi: 10.4161/23723556.2014.995047 PMC4905320 27308484

[ref11] Dixon S. J. Lemberg K. M. Lamprecht M. R. Skouta R. Zaitsev E. M. Gleason C. E. . (2012). Ferroptosis: an iron-dependent form of nonapoptotic cell death. Cell 149, 1060–1072. doi: 10.1016/j.cell.2012.03.042, 22632970. PMC3367386

[ref12] Dixon S. J. Stockwell B. R. (2019). The hallmarks of ferroptosis. Annu. Rev. Cancer Biol. 3, 35–54. doi: 10.1146/annurev-cancerbio-030518-055844, 41613499. PMC12851585

[ref13] Doll S. Freitas F. P. Shah R. Aldrovandi M. da Silva M. C. Ingold I. . (2019). FSP1 is a glutathione-independent ferroptosis suppressor. Nature 575, 693–698. doi: 10.1038/s41586-019-1707-0, 31634899.

[ref14] Duncan G. J. Manesh S. B. Hilton B. J. Assinck P. Plemel J. R. Tetzlaff W. (2020). The fate and function of oligodendrocyte progenitor cells after traumatic spinal cord injury. Glia 68, 227–245. doi: 10.1002/glia.23706, 31433109.

[ref15] Fehlings M. G. Tator C. H. (1995). The relationships among the severity of spinal cord injury, residual neurological function, axon counts, and counts of retrogradely labeled neurons after experimental spinal cord injury. Exp. Neurol. 132, 220–228. doi: 10.1016/0014-4886(95)90027-6, 7789460.

[ref16] Feng Z. Min L. Chen H. Deng W. Tan M. Liu H. . (2021). Iron overload in the motor cortex induces neuronal ferroptosis following spinal cord injury. Redox Biol. 43:101984. doi: 10.1016/j.redox.2021.101984, 33933882. PMC8105676

[ref17] Garcia E. Hernández-Ayvar F. Rodríguez-Barrera R. Flores-Romero A. Borlongan C. Ibarra A. (2022). Supplementation with vitamin E, zinc, selenium, and copper re-establishes T-cell function and improves motor recovery in a rat model of spinal cord injury. Cell Transplant. 31:9636897221109884. doi: 10.1177/09636897221109884, 35808825. PMC9272473

[ref18] Ge H. Xue X. Xian J. Yuan L. Wang L. Zou Y. . (2022). Ferrostatin-1 alleviates white matter injury via decreasing ferroptosis following spinal cord injury. Mol. Neurobiol. 59, 161–176. doi: 10.1007/s12035-021-02571-y, 34635980.

[ref19] Hu R. Sun H. Zhang Q. Chen J. Wu N. Meng H. . (2012). G-protein coupled estrogen receptor 1 mediated estrogenic neuroprotection against spinal cord injury. Crit. Care Med. 40, 3230–3237. doi: 10.1097/CCM.0b013e3182657560, 22975889.

[ref20] Huang Z. Lin J. Jiang H. Lin W. Huang Z. Chen J. . (2023). Metformin promotes Schwann cell remyelination, preserves neural tissue and improves functional recovery after spinal cord injury. Neuropeptides 100:102348. doi: 10.1016/j.npep.2023.102348, 37236132.

[ref21] Ingold I. Berndt C. Schmitt S. Doll S. Poschmann G. Buday K. . (2018). Selenium utilization by GPX4 is required to prevent hydroperoxide-induced ferroptosis. Cell 172, 409–422.e21. doi: 10.1016/j.cell.2017.11.048, 29290465.

[ref22] Jhelum P. Santos-Nogueira E. Teo W. Haumont A. Lenoël I. Stys P. K. . (2020). Ferroptosis mediates cuprizone-induced loss of oligodendrocytes and demyelination. J. Neurosci. 40, 9327–9341. doi: 10.1523/JNEUROSCI.1749-20.2020, 33106352. PMC7687057

[ref23] Kenny E. M. Fidan E. Yang Q. Anthonymuthu T. S. New L. A. Meyer E. A. . (2019). Ferroptosis contributes to neuronal death and functional outcome after traumatic brain injury. Crit. Care Med. 47, 410–418. doi: 10.1097/CCM.0000000000003555, 30531185. PMC6449247

[ref24] Kim S. Kang S. Joo J. Han S. H. Shin H. Nam B. Y. . (2021). Characterization of ferroptosis in kidney tubular cell death under diabetic conditions. Cell Death Dis. 12:160. doi: 10.1038/s41419-021-03452-x, 33558472. PMC7870666

[ref25] Kozlowski P. Raj D. Liu J. Lam C. Yung A. C. Tetzlaff W. (2008). Characterizing white matter damage in rat spinal cord with quantitative MRI and histology. J. Neurotrauma 25, 653–676. doi: 10.1089/neu.2007.0462, 18578635.

[ref26] Kraft V. A. N. Bezjian C. T. Pfeiffer S. Ringelstetter L. Müller C. Zandkarimi F. . (2020). GTP cyclohydrolase 1/tetrahydrobiopterin counteract ferroptosis through lipid remodeling. ACS Cent. Sci. 6, 41–53. doi: 10.1021/acscentsci.9b01063, 31989025. PMC6978838

[ref27] Lee H. Zandkarimi F. Zhang Y. Meena J. K. Kim J. Zhuang L. . (2020). Energy-stress-mediated AMPK activation inhibits ferroptosis. Nat. Cell Biol. 22, 225–234. doi: 10.1038/s41556-020-0461-8, 32029897. PMC7008777

[ref28] Li Y. Jin C. Shen M. Wang Z. Tan S. Chen A. . (2020). Iron regulatory protein 2 is required for artemether-mediated anti-hepatic fibrosis through ferroptosis pathway. Free Radic. Biol. Med. 160, 845–859. doi: 10.1016/j.freeradbiomed.2020.09.008, 32947011.

[ref29] Li L. Xiong Z. Qian Z. M. Zhao T. Feng H. Hu S. . (2014). Complement C5a is detrimental to histological and functional locomotor recovery after spinal cord injury in mice. Neurobiol. Dis. 66, 74–82. doi: 10.1016/j.nbd.2014.02.008, 24607885.

[ref30] Mehta S. L. Kumari S. Mendelev N. Li P. A. (2012). Selenium preserves mitochondrial function, stimulates mitochondrial biogenesis, and reduces infarct volume after focal cerebral ischemia. BMC Neurosci. 13:79. doi: 10.1186/1471-2202-13-79, 22776356. PMC3411431

[ref31] Pukos N. Goodus M. T. Sahinkaya F. R. McTigue D. H. (2019). Myelin status and oligodendrocyte lineage cells over time after spinal cord injury: what do we know and what still needs to be unwrapped? Glia 67, 2178–2202. doi: 10.1002/glia.23702, 31444938. PMC7217327

[ref32] Rayman M. P. (2012). Selenium and human health. Lancet 379, 1256–1268. doi: 10.1016/S0140-6736(12)61452-9 22381456

[ref33] Rosenblum W. I. Nelson G. H. Murata S. (1995). Protein synthesis and rapid recovery of endothelium-dependent dilation after endothelial injury of pial arterioles. Am. J. Physiol. 268, H512–H515. doi: 10.1152/ajpheart.1995.268.1.H512, 7530925.

[ref34] Schucht P. Raineteau O. Schwab M. E. Fouad K. (2002). Anatomical correlates of locomotor recovery following dorsal and ventral lesions of the rat spinal cord. Exp. Neurol. 176, 143–153. doi: 10.1006/exnr.2002.7909, 12093091.

[ref35] Seelig J. Heller R. A. Hackler J. Haubruck P. Moghaddam A. Biglari B. . (2020). Selenium and copper status—potential signposts for neurological remission after traumatic spinal cord injury. J. Trace Elem. Med Biol. 57:126415. doi: 10.1016/j.jtemb.2019.126415, 31685353.

[ref36] Seibt T. M. Proneth B. Conrad M. (2019). Role of GPX4 in ferroptosis and its pharmacological implication. Free Radic. Biol. Med. 133, 144–152. doi: 10.1016/j.freeradbiomed.2018.09.014 30219704

[ref37] Shi J. Tang R. Zhou Y. Xian J. Zuo C. Wang L. . (2020). Attenuation of white matter damage following Deferoxamine treatment in rats after spinal cord injury. World Neurosurg. 137, e9–e17. doi: 10.1016/j.wneu.2019.08.246, 31518742.

[ref38] Shi J. Xue X. Yuan L. He G. Jiang Z. Wang L. . (2023). Amelioration of white matter injury through mitigating ferroptosis following hepcidin treatment after spinal cord injury. Mol. Neurobiol. 60, 3365–3378. doi: 10.1007/s12035-023-03287-x, 36853431.

[ref39] Simons M. Nave K. A. (2015). Oligodendrocytes: myelination and axonal support. Cold Spring Harb. Perspect. Biol. 8:a020479. doi: 10.1101/cshperspect.a020479, 26101081. PMC4691794

[ref40] Xia Y. Yan Y. Xia H. Zhao T. Chu W. Hu S. . (2015). Antisense vimentin cDNA combined with chondroitinase ABC promotes axon regeneration and functional recovery following spinal cord injury in rats. Neurosci. Lett. 590, 74–79. doi: 10.1016/j.neulet.2015.01.073, 25641132.

[ref41] Xia Y. Zhao T. Li J. Li L. Hu R. Hu S. . (2008). Antisense vimentin cDNA combined with chondroitinase ABC reduces glial scar and cystic cavity formation following spinal cord injury in rats. Biochem. Biophys. Res. Commun. 377, 562–566. doi: 10.1016/j.bbrc.2008.10.024, 18930033.

[ref42] Xue P. Zhuang H. Bai T. Zeng X. Deng J. Shao S. . (2024). Iron (II)-based metal-organic framework nanozyme for boosting tumor ferroptosis through inhibiting DNA damage repair and system Xc−. J. Nanobiotechnology 22:228. doi: 10.1186/s12951-024-02508-2, 38715049. PMC11077818

[ref43] Yang W. S. SriRamaratnam R. Welsch M. E. Shimada K. Skouta R. Viswanathan V. S. . (2014). Regulation of ferroptotic cancer cell death by GPX4. Cell 156, 317–331. doi: 10.1016/j.cell.2013.12.010, 24439385. PMC4076414

[ref44] Yao X. Zhang Y. Hao J. Duan H. Zhao C. Sun C. . (2019). Deferoxamine promotes recovery of traumatic spinal cord injury by inhibiting ferroptosis. Neural Regen. Res. 14, 532–541. doi: 10.4103/1673-5374.245480, 30539824. PMC6334606

[ref45] Yeo J. E. Kim J. H. Kang S. K. (2008). Selenium attenuates ROS-mediated apoptotic cell death of injured spinal cord through prevention of mitochondria dysfunction; in vitro and in vivo study. Cell. Physiol. Biochem. 21, 225–238. doi: 10.1159/000113764, 18209489.

[ref46] Yuan J. Liu W. Zhu H. Chen Y. Zhang X. Li L. . (2017). Curcumin inhibits glial scar formation by suppressing astrocyte-induced inflammation and fibrosis in vitro and in vivo. Brain Res. 1655, 90–103. doi: 10.1016/j.brainres.2016.11.002, 27865778.

[ref47] Zhang Z. Wu Y. Yuan S. Zhang P. Zhang J. Li H. . (2018). Glutathione peroxidase 4 participates in secondary brain injury through mediating ferroptosis in a rat model of intracerebral hemorrhage. Brain Res. 1701, 112–125. doi: 10.1016/j.brainres.2018.09.012, 30205109.

[ref48] Zille M. Karuppagounder S. S. Chen Y. Gough P. J. Bertin J. Finger J. . (2017). Neuronal death after hemorrhagic stroke in vitro and in vivo shares features of ferroptosis and necroptosis. Stroke 48, 1033–1043. doi: 10.1161/STROKEAHA.116.015609, 28250197. PMC5613764

